# 

**Published:** 1914-08-15

**Authors:** 


### ANNOUNCEMENTS.

### Illinois State Dental Society.

Officers of the Illinois State Dental Society for the ensuing year, and the place of meeting selected for 1915.

President, J. M. Barcus, Carlinville.

Vice-President, J. P. Buckley, Chicago.

Secretary, Henry L. Whipple, Quincy.

Treasurer, T. P. Donelan, Springfield.

Librarian, J. D. Wilson, Danville.

The fifty-first annual meeting to be held at Peoria, May 11, 12, 13, 14, 1915. I am

Henry L. Whipple, Secretary.

Dr. Samuel T. Kirk of Kokomo, Ind., died on April 12, 1914, of diabetes, in his seventy-sixth year. Deceased was a member of the Indiana State Dental Society, and one of the founders of the Indiana Dental College at Indianapolis.

### The Arizona Dental Examiners.

The fall session of this Board will be held in Phoenix, October 5, 1914.

J. H. Blaine, Secy.

### Northern Indiana Dental Society.

The next annual meeting of the Northern Indiana Dental Society will be held August 28 and 29 at Culver (Lake Maxinkuckee.)

O. A. Van Kirk, Secretary,
Kendalville, Ind.

---

### Meeting of the Alumni Association of the Dental College of the State University of Iowa.
### Oct. 22, 23 and 24.

The next meeting of the Alumni Association of the Dental College of the State University of Iowa will be held at Iowa City during the Homecoming week on Thursday, Oct. 22, Friday, Oct. 23 and Saturday morning, Oct. 24. The Iowa-Minnesota Football game will be played Saturday afternoon, Oct. 24.

The entire meeting will be devoted to Prosthodontia. The officers take pleasure in announcing that they have secured the services of Dr. G. H. Wilson of Cleveland, Ohio, author of the most modern and thorough work on Dental Prosthetics, and Dr. Forrest H. Orton of St. Paul, Minn., professor of Crown and Bridge work at the Dental College of the State University of Minnesota.

Dr. George H. Wilson will lecture and demonstrate on Anatomical Occlusion, its underlying principles, elucidating and comparing modern methods; also the retention of Artificial Dentures, discussing the different principles and demonstrating the different methods. Dr. F. H. Orton will give a lecture and clinic on Crown and Bridge work.

Names of other prominent Prosthodontists who will contribute to this meeting will be announced later.

Richard Summa, President.
John Voss, Secretary.

